# Effect of Diet Consistency on Rat Mandibular Growth: A Geometric Morphometric and Linear Cephalometric Study

**DOI:** 10.3390/biology11060901

**Published:** 2022-06-11

**Authors:** Ioannis A. Tsolakis, Christos Verikokos, Despoina Perrea, Konstantina Alexiou, Sotiria Gizani, Apostolos I. Tsolakis

**Affiliations:** 1 Department of Orthodontics, School of Dentistry, Aristotle University of Thessaloniki, 54124 Thessaloniki, Greece; 2 Second Department of Surgery, “Laikon Hospital”, School of Medicine, National and Kapodistrian University of Athens, 11527 Athens, Greece; ch_verikokos@yahoo.com; 3 Laboratory of Experimental Surgery and Surgical Research “N.S. Christeas”, School of Medicine, National and Kapodistrian University of Athens, 15772 Athens, Greece; dperrea@med.uoa.gr; 4 Department of Oral and Maxillofacial Radiology, School of Dentistry, National and Kapodistrian University of Athens, 10679 Athens, Greece; kalexiou20@gmail.com; 5 Department of Paediatric Dentistry, Dental School, National and Kapodistrian University of Athens, 11527 Athens, Greece; sotiriagizani@gmail.com; 6 Department of Orthodontics, School of Dentistry, National and Kapodistrian University of Athens, 11527 Athens, Greece; apostso@otenet.gr; 7 Department of Orthodontics, Case Western Reserve University, Cleveland, OH 44106, USA

**Keywords:** growth, soft diet, hard diet, mandible

## Abstract

**Simple Summary:**

Craniofacial growth is affected by different environmental factors. One of these factors that seems to affect mandibulofacial growth is mastication. Diet consistency leads to different masticatory forces during mastication. Various researchers looked over the effect of diet consistency on craniofacial growth and more specifically on the mandible. The question of how diet consistency affects mandibular growth is still controversial since various studies had different results on that aspect. The results of this study support that there is a major effect of diet consistency on mandibular morphology. Thus, it contributes to a better understanding of evolution of mandibular growth.

**Abstract:**

Background: Our study intended to investigate the null hypothesis that there is no effect of diet consistency on rat mandibular growth. Methods: A total sample of 24 female wistar rats, 30 days old, was used in this study. In the first group, the rats were fed soft diet and in the second group, they were fed hard diet for 60 days. On the 60th day, the rats were sedated and lateral cephalometric X-rays were taken. Lateral cephalometric X-rays were digitized with 7 craniofacial landmarks for the linear measurements, as well as with 12 curves and 90 landmarks, of which 74 were semilandmarks and 16 were fixed landmarks for morphometric analysis. These landmarks were exposed to Procrustes superimposition and Principal Component Analysis (PCA) to describe the shape variability of the mandible. Results: Means measurements of the soft diet group compared to those of the hard diet group were significantly different in linear and morphometric analysis measurements. The soft diet group of wistar rats revealed significant changes on the condyle (smaller), the angle of the mandible, and on the body of the mandible. Conclusions: Diet consistency affects the craniofacial growth of rats. Soft diet could be responsible for less mandibular growth.

## 1. Introduction

It is well accepted that craniofacial growth is affected by environmental factors [1,2]. Moreover, the muscular loading forces play an important role on bone growth and development. Therefore mandibulofacial growth is closely associated with the movements of the jaws and loading of the orofacial region [3]. As an environmental factor, mastication seems to be liable for a spread of developmental changes within the stomatognathic system [4,5]. This may be the reasoning explanation for the mean increase of malocclusions in industrial societies in the 20th Century [6,7].

It has been noticed that many civilized human populations have been developed more severe malocclusions than the ones they had presented under primitive conditions in life. Therefore the impact of diet consistency on craniofacial growth triggers lots of research. Various experimental studies, mainly in wistar rats, studied the effect of diet consistency to craniofacial growth [8,9,10,11,12,13,14,15,16]. During the last century, the human masticatory muscles demands seem to be reduced. Modern dietary habits are acknowledged as contributors to the increased frequency of malocclusion [17,18,19]. The period of wistar rats’ rapid growth is appeared up to 5 weeks, while the growth is slowing down between 8 and 16 weeks. An increase in cell death or a decrease in proliferation could explain this decrease. Rats reach the end of their growth after 28–30 weeks [20]. In addition, phenomena that appear identical in their form may be directed by different mechanisms at different ages. This means that while the cell actions might be different, they result in identical forms [21].

Odman et al., reported that a period of 7 months with low masticatory needs in the soft diet group group during adolescence and early adulthood resulted to a smaller mandible. Morphometric analysis indicated significant differences like the area of the angular process and therefore the inclination of the condylar process [22]. In 2007 Tanaka et al. found that trabecular bone had a higher mineralization degree than cortical bone. In the anterior mandibular area, higher mineralization levels were found than those in the posterior mandibular area. In those two areas, the soft diet group appeared tp have a higher degree of mineralization than the hard diet group. The trabecular bone in the condyle of the hard diet group showed a significantly greater degree of mineralization than in the soft diet group [23]. A few years later Grunheid et al. had an equivalent hypothesis but they concluded that a moderate reduction in masticatory functional load doesn’t significantly affect the remodeling rate and therefore the degree of mineralization in areas of the mandible that are loaded during mastication but might induce a more heterogeneous mineral distribution [24].

The question of how diet consistency affects mandibular growth is still controversial since various studies had different results on that aspect. There is no study so far that looked for possible mandibular changes by using both linear and geometric morphometric analysis in order to evaluate the anatomical changes. The aim of this study is to evaluate if food consistency affects jaw growth.

## 2. Materials and Methods

An Institutional Review Board approval was obtained prior to the start of this study. The research took place at the Laboratory of Experimental Surgery and Surgical Research, National and Kapodistrian University of Athens, School of Medicine, Athens, Greece. A total sample of 24 female wistar rats aged 30 days and equally weighted (67 ± 3 g) was used in this study. They were computer-generated randomized and equally separated into two groups composed of 12 females each.

### 2.1. Diet

In the first group, the wistar rats were fed a soft diet and in the second group, they were fed a hard diet for 60 days. The hard diet groups were fed the ordinary diet for rats in hard pellet form (R34; Lactamin). The soft diet groups received the ordinary diet, ground and mixed with water in standardized proportions (2 parts food:5 parts water) that was left for 24 h in order for every particle to be soft. As a result, both groups received the same nutrition with a different consistency. The bedding material of the cages of this group was sifted to exclude large particles that could stimulate extra gnawing activity.

### 2.2. Radiograph Acquisition

On the 60th day, lateral X-rays were taken. Cephalometric X-rays were chosen to be taken after 60 days of experimentation because it is well documented that a higher percentage of mandibular growth takes part during the second and third month of rats’ life.

Before the X-ray was performed, all wistar rats went under a weight measuring procedure. A special cephalostat was designed as it was firstly described by Tsolakis et al. [25]. The cephalostat for our experiment was a Plexiglas base with vertical projections, where holes were drilled at 5 mm intervals, and two ear posts were attached. A vertical extension at the distal edge of the Plexiglas part supported the entire component on a base bearing a vertical extension. There was another horizontal component mounted on the top of the extension at a 90 degree angle, attached to a hanger at its end from which the animal was suspended from the upper anterior teeth by string. By means of their upper incisors, the animals were suspended from the horizontal post. The ear posts were set in a position corresponding to the animal’s earholes at the plastic vertical projections. The X-ray machine was placed at a distance of 7 feet from the cephalostat. We used the Siemens Nanodor1 X-ray machine and the Carestream Insight No4 (3.1 × 4.4 cm) X-ray film. The Siemens X-ray machine was adjusted to 15 milliampers and 75 kilovolts. The animals were exposed to radiation for 0.8 s. All the animals were sedated by injecting 25 mL of Rompun and 100 mL of Imalgen in order to be stable during the procedure. Manually developed X-ray films took five minutes to develop and eight minutes to fix. Following fixation, the films were washed for 1 h and air-dried. In order to digitize the X-rays, we scanned them by using the Epson V750 Pro. Once the films were scanned they were uploaded to the Viewbox software.

### 2.3. Data Processing

**Figure 1:**
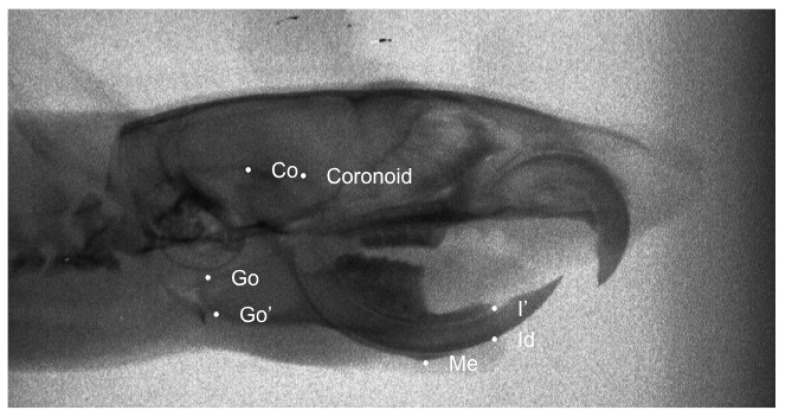
Traced X-ray for linear mesasurement. The landmarks we used are the most posterior-superior point on the mandibular condyle (Co), the most posterior point of the angular process of the mandible (Go), the point on the most inferior contour of the angular process of the mandible (Go’), the most posterosuperior point of condylar process (Coronoid), the most inferior and anterior point of the lower border of the mandible (Me), the most inferior and anterior point on the alveolar process of the mandible (Id) and the most anterior edge of the alveolar bone on the convexity of the lower incisor (I’).

**Figure 2:**
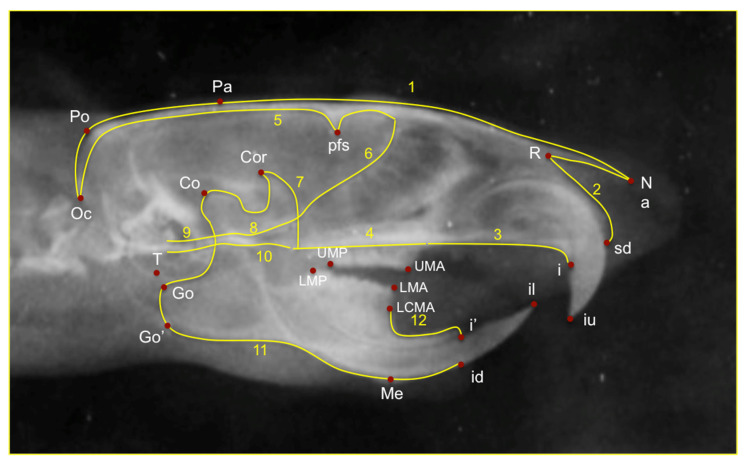
Traced lateral X-ray for Geometric morphometric analysis. 12 curves and 90 landmarks, of which 74 were semilandmarks and 16 were fixed landmarks were used for morphometric analysis. The fixed landmarks are the most posterior-superior point on the mandibular condyle (Co), the most posterior point of the angular process of the mandible (Go), the point on the most inferior contour of the angular process of the mandible (Go’), most prominent point between incisal edges of lower incisors (il), most prominent point between incisal edges of upper incisors (iu), most posterior point of lower molars (LMP), most anterior point of lower molars (LMA), the most inferior-anterior point of the lower border of the mandible (Me), the most posterior point of squama occipitalis (Oc), the most superior point of parietal bone (Pa), the internal curvature of the frontal bone (pfs), the point corresponding to anatomic porium (Po), The deepest point of the nasopremaxillary suture (R), the most inferior point of tympanic bone (T), the most posterior edge of the alveolar bone on the convexity of the upper incisors (i) and the most inferior-anterior point on the alveolar process of premaxilla (sd).

The main craniofacial structures depicted on lateral cephalograms were digitized and traced by a blinded operator with 12 curves and 90 landmarks, of which 74 were semilandmarks and 16 were fixed landmarks for morphometric analysis. These landmarks were subjected to Procrustes superimposition and Principal Component Analysis (PCA) in order to describe shape variability of the cranial base, maxilla, and mandible, as well as of the whole craniofacial complex. Seven landmarks were selected for the linear measurements on the lateral X-rays. (Table 1). Once the X-rays were uploaded linear and geometric morphometric measurements were performed. (Figure 1 and Figure 2). The linear measurements of choice were differentiated into those that measured the length of the mandible and those that measured the posterior height of the mandible (Table 2).

### 2.4. Statistical Analysis

The number of animals in each group should be low and should be the minimum to obtain statistically reliable results. Power analysis was used in order to estimate the number of animals with a confidence level of 90% based on previous relevant studies. The operator’s reliability was calculated using intraclass correlation on 12 randomly selected animals, whose data was re-measured 3 weeks apart. Paired *t*-test was performed for the weight analysis. Differences related to diet were assessed using regression analyses. Each measurement was regressed on diet and their interaction. Shapiro-Wilk test was used to assess the normality of the sample. When the normality assumption for the residuals was violated, quantile regression was used. Comparisons were adjusted using the Bonferroni method for multiple comparisons. Analysis was performed on α = 5% level of statistical significance. For statistical analysis of morphometric measurements, a permutation test was used (10,000 permutations without replacement).

## 3. Results

### 3.1. Weight Measurement

There were no statistically significant differences between the two groups for the weight measurements. (*p* = 0.074) (Table 3).

### 3.2. Geometric Morphometric Analysis

**Figure 3:**
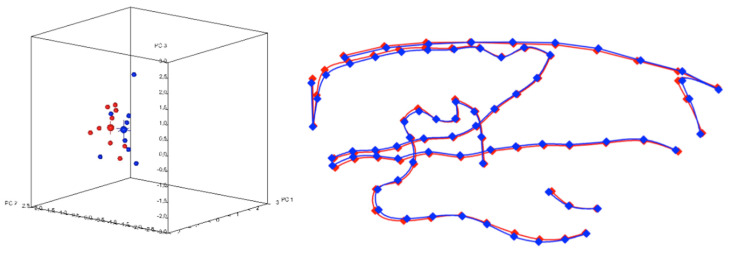
Plot of sample and corresponding superimposition of the mean tracings of the two groups. Blue tracing corresponds to soft diet group and red tracing corresponds to hard diet group.

The first three principal components (PC1, PC2, PC3) accounted for 55.7% of the sample’s variability and described variability in the vertical direction (hypodivergent or hyperdivergent skeletal pattern), in the anteroposterior direction (orthognathia, retrogna thia, prognathia) and in the gonial angle of the mandible (low gonial angle or high gonial angle), respectively. The permutation test on the morphometric measurements showed significant differences between the soft and hard diet groups. (*p* = 0.002). A regional Pro crustes superimposition of the average shapes of wistar rats was performed in order to visualize these differences. The superimposition of the morphometric means of each group showed differences in the condyle, the angle of the mandible, and the body of the mandible (Figure 3).

### 3.3. Length Measurement

The linear measurements that indicated the differences on mandibular length were Go’-Me, Go-Me, Coronoid-Me, Co-Me, Co-Id, Co-I’. As shown in Table 4, there were statistically significant differences between all soft diet groups when they were compared with the hard diet groups in all linear measurements. All measurements were highly reliable according to the reliability test (Table 4 and Table 5).

### 3.4. Posterior Height Measurement

The indications for the posterior mandibular height were Co-Go, Co-Go’ linear measurements. There were statistically significant differences between the soft diet group and the hard diet group in all linear measurements. All measurements were highly reliable according to the reliability test (Table 4 and Table 5).

## 4. Discussion

In our study twenty-four wistar rats were grouped into hard diet group and soft diet group. Wistar rats were the experimental animal of choice because they are small, easy to house, report a minimal social concern, are short-lived and have well-known genetics, closely related to human development [12,13,14,15,16]. We included only female rats in our study in order to minimize any possible bias. It is worthy to mention that growth patterns are not differentiated due to different gender. After 60 days of experimentation, lateral cephalometric X-rays were taken. We investigated the effects of dietary physical consistency on craniofacial growth, using lateral cephalometric analysis as well as some of the previous studies. According to the linear measurements, the significant differences between the hard diet and soft diet groups were determined at 7 measuring points. We found statistically significant differences in all linear measurements between the soft diet group and the hard diet group. Those results indicated that diet consistency could possibly affect the growth of the mandible. The length and the posterior height of the mandible seem to get smaller when the diet is more likely soft. According to the geometric morphometric measurements, there were significant differences between the two groups. The superimposition of the morphometric means indicates that there are differences in the condyle, the angle of the mandible, and the body of the mandible. It is important to mention that we could not identify macroscopically any excessive teeth growth or other modification.

Craniofacial growth and development have been studied by various scientists. Anthropologists and Orthodontists were the main scientists that looked over the evolution of the skull, maxilla, and mandible. It has been found that the relationship between the two jaws was changing over the decades. Later on, it was proved by Orthodontists and other medical fields that a retrognathic mandible could result in airway obstruction, mastication discomfort, and dysfunction in the temporomandibular joint leading to TMDs. The relationship of the two jaws could affect the entire craniofacial growth since all these results will have a general health impact.

In 1999 Kiliaridis et al., concluded that a low masticatory function directs to reduced growth of the condyle using morphometric analysis [26]. This may be the result of an alteration within the stress distribution in the temporomandibular joint area, due to the lack of extensive masticatory forces. Nine years after, Odman et al., found that the area of the mandible was smaller in the soft diet group compared to the normal group [12]. Through morphometric analysis, they found only marginal differences of the adult rat mandibular morphology during a 6-week period of masticatory function rehabilitation. However, the observed catch-up trend might indicate that an extended rehabilitation period may have a substantial impact on mandibular morphology.

There were previous studies that looked at those differences through cephalometric analysis. Killiaridis et al., found that the growth rate in the gonial angle of mandible was increased in the hard diet groups but the angle between occlusal and mandibular plane was reduced in the hard diet groups [27]. In 2002 Maki et al., showed the ramus height and the height of the condylar process and coronoid process were increased in the hard diet groups. According to their research, there was not any substantial difference between hard diet and soft diet groups in the mandibular length [28]. Abed et al. (2007) reported that the anterior corpus length, the ramus height, the bigonial width were increased in the hard diet groups [29]. Hichijo et al., agreed with the previous studies that the ramus height was increased for the hard diet groups. The ramus angle and the gonial angle were decreased in the hard diet groups. The mandibular length, the mandibular base length, and the height of the coronoid process demonstrated no significant difference between hard diet and soft diet groups [30].

This study investigated the influence of diet consistency on mandibular growth. This is the only study to our knowledge that used both linear and geometric morphometric analysis on the same sample to measure the growth changes. A summary of this research is reported in the table of the ARRIVE guidelines. (Table 6). The limitation of this study could possibly be the duration of the experiment since the rat craniofacial region grows during the first four months of life. Most of the previous studies looked over 30 days of growth changes but there might be even more changes on the mandible during the later days of growth. Another limitation could probably be the 2D imaging of the wistar rats’ heads. In this way, we are missing the transversal changes in craniofacial morphology that may occur during growth. Lastly, a limitation of our study is the analog X-ray system that was used. Nevertheless, all X-rays were manually developed by the same scientist and they were scanned to obtain digital images by the same scientist also. In this way any possible systematic errors could influence equally the X-ray experimental procedure.

## 5. Conclusions

In conclusion, the results of this study indicate that diet consistency has a significant influence on craniofacial growth and development. There is a major sagittal and vertical development of the mandible of rats fed with a hard- diet than a soft-consistency diet. Moreover diet consistency influences mandibular morphology including the condylar area, the angle of the mandible and the body of the mandible.

## Figures and Tables

**Table 1:** Cephalometric landmarks used for linear measurements.

Cephalometric Landmarks	Definition
Co	Most posterior-superior point on the mandibular condyle.
Go	Most posterior point of the angular process of the mandible
Go’	Point on the most inferior contour of the angular process of the mandible
Coronoid	Most posterosuperior point of condylar process
Me	The most inferior and anterior point of the lower border of the mandible
Id	Most inferior and anterior point on the alveolar process of the mandible
I’	The most anterior edge of the alveolar bone on the convexity of the lower incisor.

**Table 2:** Linear cephalometric measurements corresponding to mandibular length and height.

Structures	Cephalometric Measurements
Mandibular length	Co-Me
	Coronoid-Me
	Go-Me
	Go’-Me
	Co-Id
	Co-I’
Posterior mandibular height	Co-Go
	Co-Go’

**Table 3:** Weight measurement results.

	Mean Weight (g)	SD	*p* Value
Soft Diet	248.4	5.1	*p* = 0.074
Hard Diet	247.7	5.5	

**Table 4:** Reliability test performed on 10 randomly selected subjects re-measured 3 weeks apart.

Variables		Cochran’s Alpha
Linear measurements	Go’-Me	0.833
	Go-Me	0.857
	Coronoid-Me	0.859
	Co-Me	0.822
	Co-Id	0.934
	Co-I’	0.944
	Co-Go	0.923
	Co-Go’	0.911

**Table 5:** Mean values, standard deviations and *p* values for all linear measurements.

Linear Measurements	Diet S	Diet H	
	(n = 12)	(n = 12)	
	Mean (SD)	Mean (SD)	S-H
Go’-Me	15.80 (1.86)	18.65 (1.28)	**<0.001**
Go-Me	17.38 (1.32)	20.59 (1.42)	**<0.001**
Coronoid-Me	13.98 (1.11)	15.74 (1.42)	**0.001**
Co-Me	17.43 (1.24)	20.74 (1.56)	**<0.001**
Co-Id	20.59 (1.17)	24.47 (1.53)	**<0.001**
Co-I’	19.84 (1.16)	23.42 (1.47)	**<0.001**
Co-Go	5.91 (0.49)	7.65 (0.55)	**<0.001**
Co-Go’	6.97 (0.69)	8.84 (0.61)	**<0.001**

**Table 6:** ARRIVE guidelines.

**Study design**	1	a. In the first group the female Wistar were fed a soft diet and in the second group, they were fed a hard diet b. Animals were kept in separate cages. The bedding material of the cages of this group was sifted to exclude large particles that could stimulate extra gnawing activity. Page 2,3
**Sample size**	2	a. A total sample of 24 female Wistar aged 30 days was used in this study. (12 for each group) b. Sample size was decided according to previous similar research. Page 2
**Inclusion and** **exclusion criteria**	3	a. The soft diet group received the ordinary diet, ground and mixed with water in standardized proportions. The hard diet groups were fed the ordinary diet for rats in hard pellet form b. Soft diet group (n = 12), Hard diet group (n = 12) Page 2,3
**Randomization**	4	a. They were computer-generated randomized and equally separated into two groups Page 2
**Blinding**	5	a. The operator for linear and geometric morphometric analysis was blinded. It was given a random number for each wistar that only the rest of the authors knew to which group corresponds. Page 3
**Outcome** **measures**	6	a. Mandibular morphology Page 4
**Statistical methods**	7	a. Differences related to diet, were assessed using regression analyses. Each measurement was regressed on diet and their interaction. When the normality assumption for the residuals was violated, quantile regression was used. Comparisons were adjusted using the Bonferroni method for multiple comparisons. Analysis was performed on α = 5% level of statistical significance. For statistical analysis of morphometric measurements, a permutation test was used (10,000 permutations without replacement). Page 3
**Experimental animals**	8	a. Female Wistar rats b. Healthy female wistar rats Page 2
**Experimental procedures**	9	a. Each group was fed daily for a 30 days period time with the appropriate diet. b. Afterwards the sedated animals were hung via their upper incisors from the horizontal post and the ear posts were placed in a position corresponding to the animal’s earholes at the plastic vertical projections. c. The X-ray films were manually developed with a developing time of 5 min and a fixing time of 8 min. The films were washed for 1 h after fixation and air-dried. d. In order to digitize the X-rays, we scanned them by using the Epson V750 Pro. e. Once the films were scanned they were uploaded to the Viewbox software and then digitally traced. Page 2,3
**Results**	10	a. They were significant differences between all soft diet groups when they were compared with the hard diet groups in all linear measurements. (Go’-Me, Go-Me, Coronoid-Me, Co-Me, Co-Id, Co-I’, Co-Go, Co-Go’) b. The superimposition of the morphometric means of each group showed differences on the condyle, the angle of the mandible, and the body of the mandible. Page 5,6

## Data Availability

The datasets used and/or analyzed during the current study are available from the corresponding author upon reasonable request.
